# Sedation with dexmedetomidine is associated with transient gallbladder wall thickening and peritoneal effusion in some dogs undergoing abdominal ultrasonography

**DOI:** 10.1111/jvim.16306

**Published:** 2021-11-11

**Authors:** Marc A. Seitz, Alison M. Lee, Kimberly A. Woodruff, Alexis C. Thompson

**Affiliations:** ^1^ Department of Clinical Sciences, College of Veterinary Medicine Mississippi State University Mississippi State Mississippi USA; ^2^ Department of Comparative Biomedical Sciences, College of Veterinary Medicine Mississippi State University Mississippi State Mississippi USA

**Keywords:** A‐FAST, dexdomitor, edema, halo, hepatobiliary

## Abstract

**Background:**

Dexmedetomidine often is used for sedation before or during abdominal ultrasonography. The effect of dexmedetomidine on gallbladder wall thickness is unknown.

**Hypothesis/Objectives:**

To investigate the relationship between dexmedetomidine administration and gallbladder wall thickening in dogs. The hypothesis was that sedation with dexmedetomidine will cause transient gallbladder wall thickening. Gallbladder wall thickness will be associated with duration of sedation and recumbency position.

**Animals:**

Seventy‐nine client owned dogs and 10 healthy research dogs.

**Methods:**

A prospective observational study (n = 79) was used to establish the prevalence of gallbladder wall thickening (> 2.0 mm) after sedation with dexmedetomidine. A randomized, crossover study (n = 10) was used to evaluate the effect of time and recumbency position on the development of gallbladder wall thickening. Linear mixed models were used.

**Results:**

The proportion of client‐owned dogs that developed gallbladder wall thickening was 24.05% (19/79; 95% confidence interval [CI], 15.1%‐35.0%) with a median dose of dexmedetomidine of 5.0 μg/kg (range, 2.0‐12.5 μg/kg). After sedation, the proportion of research dogs that developed gallbladder wall thickening in left lateral (5/10, 50%; 95% CI, 18.7%‐81.3%) and dorsal (7/10, 70%; 95% CI, 34.8%‐93.3%) recumbency did not differ significantly (*P* = .45). Gallbladder wall thickening developed within 20 to 40 minutes. Duration of sedation was significantly associated with thickening of the gallbladder wall (*P* < .001). Five dogs developed 9 instances of peritoneal effusion in both lateral (5) and dorsal (4) recumbency.

**Conclusions and Clinical Importance:**

Sedation with dexmedetomidine is associated with gallbladder wall thickening (> 2.0 mm) and peritoneal effusion that could be confused with pathologic etiologies.

**Abbreviations**
AO: aorta
BCS: body condition score
CI: confidence interval
PBFV: portal blood flow volume
PV: portal vein

## 1 INTRODUCTION

Ultrasonography is commonly used to evaluate the hepatobiliary system in dogs. The normal gallbladder wall in dogs is visible ultrasonographically as a thin hyperechoic line measuring 1 to 2 mm thick.^1^ Gallbladder wall thickening is often the result of gallbladder wall edema and most commonly appears as a hypoechoic band of variable thickness surrounded by inner and outer hyperechoic lines.^2^ This appearance has been referred to as a “double rim sign” or a “gallbladder halo.”^3^

Gallbladder wall edema is well established in the veterinary literature and has been reported with numerous etiologies that cause inflammation, venous congestion, or a combination of both. Examples include cholecystitis, hepatopathy, pancreatitis, hepatobiliary neoplasia, biliary obstruction, portal hypertension, right‐sided heart failure (including pericardial effusion), hypoproteinemia, chemotherapeutic drugs, renal failure, and more recently anaphylaxis, blood transfusion, immune‐mediated hemolytic anemia, sepsis, systemic inflammatory response syndrome, and disseminated intravascular coagulation.^2^, ^4^, ^5^, ^6^ The presence of peritoneal effusion surrounding the gallbladder may mimic true gallbladder wall thickening.^2^ The proposed pathophysiology of gallbladder wall edema is explained by aberrations of Starling's forces, including increased vascular permeability, increased hydrostatic pressure in the portal venous system, decreased oncotic pressure, or lymphatic obstruction.^2^, ^5^ Although gallbladder wall edema is a common cause of gallbladder wall thickening, other factors such as cystic mucosal hyperplasia or fibrosis also may contribute to the thickened appearance. A review of the literature identified no reports of gallbladder wall thickening after administration of dexmedetomidine or other alpha‐2 agonists.

Many dogs tolerate abdominal ultrasonography with only physical restraint, but periprocedural sedation often is required in noncompliant patients or for ultrasound‐guided procedures. Dexmedetomidine, an alpha‐2 agonist, often is chosen for sedation because of its reversible sedative, anxiolytic, and analgesic properties.^7^ On several occasions, we have observed variable and progressive gallbladder wall thickening during abdominal ultrasonography in dogs sedated with dexmedetomidine that was neither present before sedation nor explained by anaphylaxis or other pathology. Our purpose was to investigate the relationship between sedation with dexmedetomidine and gallbladder wall thickening in dogs as well as factors contributing to its formation. One hypothesis was that sedation with dexmedetomidine will cause transient gallbladder wall thickening in dogs. A second hypothesis was that increased gallbladder wall thickness will be associated with increasing sedation dose, a longer duration of sedation, recumbency in a dorsal position, obesity, increased congestion index, and increased portal blood flow volume (PBFV) but not associated with other secondary variables.

## 2 MATERIALS AND METHODS

Two phases were planned to investigate the study's purpose. Both phases were approved by the Mississippi State University Institutional Animal Care and Use Committee (IACUC‐19‐024 and IACUC‐19‐026). Phase 1 had a prospective observational design whereas phase 2 had a randomized, crossover design. For all‐client owned animals, owners signed an informed consent form before recruitment into phase 1.

### 2.1 Phase 1

Phase 1 was designed to establish the proportion of client‐owned dogs that develop gallbladder wall thickening (> 2.0 mm) when sedated with dexmedetomidine (IV or IM) alone or in combination with other drugs. Based on our clinical experience before the study, the estimated proportion of dogs that develop gallbladder wall thickening after being sedated with dexmedetomidine was approximately 6% to 12%. Assuming the proportion of dogs developing gallbladder wall thickening was 6% and an α level of .05, sample size calculation identified that 79 patients will allow an estimate of this proportion with precision of ±5.3%. If gallbladder wall thickening was not detected in any of the 79 dogs, then it was estimated with 95% confidence that this finding occurs at a level < 3.7%.

Dogs eligible for inclusion were those > 1 year of age that presented to the diagnostic imaging service for routine nonabdominal radiographic imaging studies that required sedation with dexmedetomidine (Dexdomitor, Zoetis, Troy Hills, New Jersey) alone or in combination with other drugs. A history, physical examination, CBC, and serum biochemical profile were performed the same day as the ultrasound examinations for all dogs. Dogs were not included if they had a history of clinical signs related to potential abdominal pathology (eg, anorexia, vomiting, diarrhea, abdominal pain), muffled heart sounds, dyspnea, increased liver function test results (eg, alanine aminotransferase, alkaline phosphatase, total bilirubin), a platelet count < 60, 000/μL, or an ultrasonographically abnormal liver, gallbladder wall thickening, or bile duct dilatation (> 3 mm) on presedation examination. Dogs also were not included in the study if they had diseases or treatments reported to cause gallbladder wall thickening, including hepatic or gallbladder disease, anaphylaxis or any other kind of shock, right‐sided heart failure, pericardial effusion, pancreatitis, hypoalbuminemia, peritoneal effusion, portal hypertension, sepsis, systemic inflammatory response syndrome, or had received fluid therapy, iodinated contrast, chemotherapeutics, sedatives, or anesthesia within 24 hours before presentation.

Dogs were sedated using a drug protocol chosen by the attending veterinarian. The following vital signs were monitored and recorded every 5 to 10 minutes: heart rate, respiratory rate, pulse quality, mucous membrane color, capillary refill time, and temperature. All dogs received a focused ultrasound examination of the liver, gallbladder, and porta hepatis before and at the end of the sedation period, and before the administration of any reversal agents (eg, atipamezole). All ultrasound examinations were performed with the dog in left lateral recumbency by the same second year radiology resident (M.A. Seitz) under the supervision of a board‐certified radiologist. The results of the presedation ultrasound examination served as an internal control and to screen for pre‐existing gallbladder wall thickening. The same ultrasound machine (LOGIQ S8, General Electric Healthcare, Chicago, Illinois) was used for all examinations. The same microconvex transducer (C3‐10 D broad‐spectrum microconvex transducer 2‐11 MHz, General Electric Healthcare) was used for all patients unless patient size limited evaluation of deeper structures because of beam attenuation. In those patients, a lower frequency probe (M5S‐D broad‐spectrum sector transducer 1‐5 MHz, General Electric Healthcare) was used. The following ultrasonographic data were recorded: gallbladder wall thickness, presence or absence of gravity‐dependent intraluminal hyperechoic gallbladder debris (sludge), and cross‐sectional area and blood flow velocity of the aorta (AO), caudal vena cava, and portal vein (PV). Blood flow velocities were measured using pulse wave Doppler with an angle of interrogation < 60°. Still images and cine loops were obtained for documentation purposes. Gallbladder wall thickening was defined as a gallbladder wall thickness > 2 mm. Each gallbladder wall and vessel cross‐sectional area were measured twice by a single observer (a radiology resident under supervision of a board‐certified veterinary radiologist) and the measurements averaged. Congestion index and PBFV were calculated as previously described.^8^ Additionally, the medical record was used to collect the following variables: final diagnosis, breed, sex, neuter status, age, weight, body condition score (BCS), sedation duration, other drugs used, and heart rate during sedation.

### 2.2 Phase 1: Statistical analysis

All data were collected in an electronic spreadsheet program (Excel, Microsoft, Redmond, Washington) and imported into a statistical software program (SAS for Windows v9.4, SAS Institute, Inc, Cary, North Carolina). Normality was evaluated using the Shapiro‐Wilk test for age, weight, BCS, dexmedetomidine dose, duration of sedation, and pre‐ and postsedation gallbladder wall thickness using the univariate procedure of the statistical software. A significant value for the Shapiro‐Wilk test indicates that the data are nonparametric. Descriptive statistics of mean, SD, median, and range were calculated, as appropriate given the distribution of the data, using the means procedure of the statistical software. Proportions and Clopper‐Pearson exact confidence interval (CI) were calculated for the number of dogs that developed gallbladder thickening using the electronic spreadsheet program. The potential association between gallbladder wall thickening and secondary measures (Table 1) was assessed in a separate logistic regression model using the logistic procedure in the statistical software program. For some secondary measures, there were no dogs with gallbladder wall thickening in 1 of the levels of the variable leading to quasi‐complete separation of data points. Firth's penalized maximum likelihood estimation was used in these models. The level of significance was set at an α level of .05.

**TABLE 1:** Summary of variables measured in phase 1 to assess their association with the formation of gallbladder wall thickening in a population of 79 client‐owned pets presenting for routine nonabdominal imaging using univariate logistic regression

Variable	n	Odds ratio	95% CI	*P* value
Sedation duration (min)	79	1.009	0.977‐1.041	.6
Dexmedetomidine dose (μg/kg)	79	0.994	0.737‐1.341	.97
Butorphanol vs other drugs	79	2.000	0.225‐17.749	.53
Gallbladder sludge (yes vs no)	79	0.889	0.313‐2.523	.82
Body weight category^b^	79			.32
Large vs small		8.996	0.434‐186.419	.16
Medium vs small		5.877	0.236‐146.635	.28
BCS category (overweight vs ideal)	79	0.680	0.240‐1.929	.47
Age category^b^	79			.12
Geriatric vs young		0.091	0.004‐1.969	.13
Middle age vs young		0.423	0.144‐1.240	.12
Breed^b^	79	n/e	n/e	.99
Average heart rate (beats per minute)	76	0.986	0.944‐1.029	.51
Presedation AO velocity (cm/s)	45	0.989	0.965‐1.014	.39
Postsedation AO velocity (cm/s)	42	0.968	0.953‐1.020	.41
Presedation CVC velocity (cm/s)	47	1.001	0.961‐1.044	.95
Postsedation CVC velocity (cm/s)	52	0.963	0.908‐1.022	.22
Presedation PV velocity (cm/s)	38	1.038	0.903‐1.192	.6
Postsedation PV velocity (cm/s)	43	0.858	0.687‐1.071	.18
Presedation congestion index (cm × s)	26	n/e	n/e	.32
Postsedation congestion index (cm × s)	34	n/e	n/e	.77
Presedation portal blood flow volume (mL/min/kg)	28	0.986	0.919‐1.058	.69
Postsedation portal blood flow volume (mL/min/kg)	35	0.808	0.669–0.976	.03^a^

Abbreviations: AO, aorta; BCS, body condition score; CI, confidence interval; CVC, caudal vena cava; n/e, not estimable; PV, portal vein.

^a^ Significant association.

^b^ Firth's penalized maximum likelihood estimation was used.

### 2.3 Phase 2

Phase 2 was designed to evaluate the effect of time and recumbency position (left lateral vs dorsal) on the development of gallbladder wall thickening in a colony of 10 healthy research dogs sedated with only 10 μg/kg of dexmedetomidine (Dexdomitor, Zoetis) administered IV. A physical examination, CBC, and serum biochemistry profile were performed ≤ 1 week before the study in all dogs. All vital signs described in phase 1 as well as noninvasive oscillometric blood pressure were monitored every 5 minutes. Dogs were randomly assigned into 1 of the 2 recumbency groups using the random number generator function of an electronic spreadsheet program. The hepatobiliary system and porta hepatis vasculature were interrogated in a manner identical to phase 1 before sedation, every 10 minutes after sedation for 90 minutes total, and 10 minutes after reversal of sedation with an equal volume of atipamezole (Antiseden, Zoetis) administered IM. Focused ultrasound examination was repeated in dogs with gallbladder wall thickening at least once every 12 to 24 hours until resolution of ultrasonographic changes. The procedure for all 10 dogs was completed within 1 week. The next week, each dog crossed over into the opposite recumbency position and the procedure was repeated ≥ 72 hours after the first procedure. The following variables were recorded similar to phase 1 for statistical analysis: body weight; heart rate; blood pressure; AO, caudal vena cava, and PV cross sectional area and blood flow velocity; presence or absence of gravity‐dependent intraluminal hyperechoic gallbladder debris (sludge); and the development of peritoneal effusion in the hepatobiliary region.

### 2.4 Phase 2: Statistical analysis

A priori power analysis that assumed an α level of .05 and a SD of 15 (calculated by assuming a range of 0‐90 minutes divided by 6) indicated a sample size of 10 dogs would allow the detection of a difference of 16 minutes with a power of 0.83. Assessment of normality, descriptive statistics, and CI were calculated as described previously for phase 1. The proportion of dogs that developed gallbladder thickening in lateral and dorsal recumbency was calculated as previously described. The effects of recumbency position and other secondary variables on gallbladder thickness were assessed separately using linear mixed models and the mixed procedure of the statistical software program. Recumbency position (the independent variable) and the interaction between position and the independent variable were included as fixed effects with Kenward Rogers degrees of freedom. A repeated statement with position within dog as the subject was used to account for the repeated measure by time. An autoregressive covariance structure was used. Dog identity was included as a random effect. If the effect of the interaction between position and the independent variable was not significant, the interaction term was removed from the model and the model re‐evaluated. For significant categorical independent variables, differences in least squares means were used to assess the effect of the variable. A similar model was used to determine the effects of recumbency position and time on gallbladder thickness with Kenward Rogers degrees of freedom and group effect of time defined to account for unequal variances across time. Visual assessment of the residuals was used to determine if the assumptions of normality and homoscedasticity were met. The level of significance was set at an α of .05.

## 3 RESULTS

### 3.1 Phase 1: Study population

Seventy‐nine client‐owned dogs were eligible for inclusion in phase 1. The study was terminated before the prestudy goal of 100 dogs because interim analysis indicated a much higher prevalence than expected and because of unanticipated recruitment challenges arising from the COVID‐19 pandemic. The study population consisted of a variety of dogs presented primarily for orthopedic conditions. Reasons for imaging included diagnosis or re‐evaluation of the following conditions: cranial cruciate ligament injury (n = 46), osteoarthrosis (8), traumatic fracture if > 24 hours after the traumatic event (5), medial patellar luxation (5), tendinopathy (4), elbow dysplasia (1), medial meniscal tear (1), osteochondritis dissecans (1), osteosarcoma (1), degenerative lumbosacral stenosis (1), cutaneous nodule (1), pododermatitis (1), ingrown nail (1), periorbital abscess (1), urinary tract infection (1), and routine wellness screening (1). Thirty‐nine dogs were female and 40 were male. Sixty‐seven dogs were neutered whereas 12 were intact. The age range was 1 to 14 years with a median of 5 years. Age was further categorized as follow: young = 1 to 4 years (33), middle‐aged = 5 to 8 years (37), and geriatric ≥ 9 (9). The following breeds were represented: mixed (14), Labrador retriever (11), English bulldog (7), Golden retriever (7), German shepherd dog (5), pit bull terrier (5), Yorkshire terrier (4), boxer (3), Pembroke welsh corgi (3), beagle (2), Catahoula (2), great dane (2), labradoodle (2), Australian shepherd (1), Brittany spaniel (1), Chesapeake bay retriever (1), collie (1), Doberman pinscher (1), maltipoo (1), maltese (1), mastiff (1), miniature pinscher (1), rottweiler (1), Shiba Inu (1), toy poodle (1). The weight range was 2.5 to 55.8 kg, with a median of 30.5 kg. Finally, BCS ranged from 3 to 8 with a median of 5. Given the variety of breeds and sizes, dogs were grouped by weight into the following categories: < 10 kg = small breed (10), 10.1 to 25 kg = medium breed (15), and > 25.1 kg = large breed (54). Body condition score also was further divided into ideal if ≤ 5 or overweight if > 5.

### 3.2 Phase 1: Results

The proportion of dogs developing gallbladder wall thickening in phase 1 was 24.1% (19/79; 95% CI, 15.1%‐35.0%) with a median dose of dexmedetomidine of 5.0 μg/kg (range, 2.0‐12.5 μg/kg) and a median duration of sedation of 30 minutes (range, 14‐92 minutes). Dogs that developed gallbladder wall thickening received a median dose of 5.0 μg/kg (range, 4.8‐10.2 μg/kg) whereas dogs that did not develop gallbladder wall thickening received a median dose of 5.0 μg/kg (range, 2.0‐12.5 μg/kg). The mean presedation gallbladder wall thickness for all dogs was 1.4 ± 0.03 mm (range, 0.7‐2.0 mm). Median postsedation gallbladder wall thickness for dogs that developed gallbladder wall thickening was 3.0 mm (range, 2.6‐4.1 mm) vs 1.3 mm (range, 0.8‐2.0 mm) for dogs that did not. Two patterns of thickening were observed—diffuse and focally asymmetric. Although not objectively quantified, thickening occurred along the dorsal gallbladder wall in most of the focally asymmetric occurrences.

Butorphanol was the drug most commonly used in combination with dexmedetomidine (72), with sporadic use of methadone (3), hydromorphone alone (3), or hydromorphone in combination with ketamine (1). During the ultrasound examinations, 35 dogs had variable degrees of gallbladder sludge whereas 44 did not. None of the following variables were significantly associated with development of gallbladder wall thickening (see Table 1): duration of sedation; dexmedetomidine dose; use of butorphanol vs other sedatives; presence of gallbladder sludge; weight category; BCS category; age category; breed; average heart rate; pre‐ or postsedation blood flow velocity of the AO, caudal vena cava, or PV; pre‐ or postsedation congestion index; or presedation PBFV. Unexpectedly, increasing PBVF was significantly associated with not developing gallbladder wall thickening (*P* = .03, odds ratio = 0.808; 95% CI, 0.669‐0.976).

### 3.3 Phase 2: Study population

A research colony of 10 healthy bloodhounds was used for phase 2. The body weight range was 22.3 to 36.1 kg, with a median of 25.1 kg. All dogs had an ideal BCS of 4 or 5. The age range was 3 to 6 years, with a median of 5 years. Seven dogs were female and 3 were male. Seven dogs were intact and 3 of the female dogs were spayed.

### 3.4 Phase 2: Results

**FIGURE 1:**
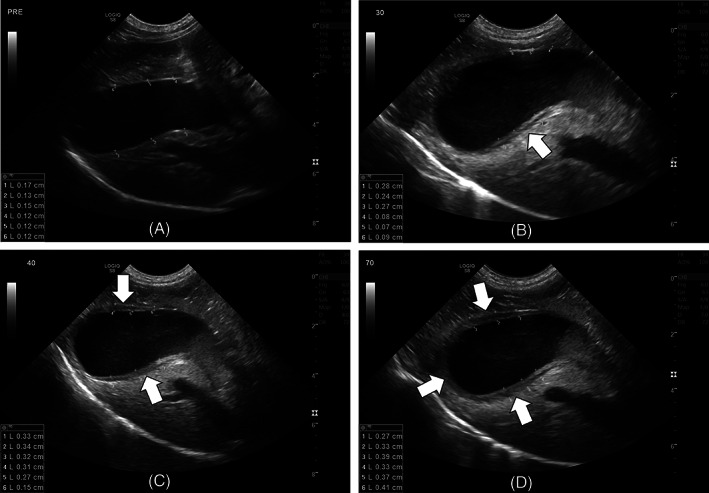
Still images from a research dog in phase 2 at presedation (A) with dexmedetomidine and 30 minutes (B), 40 minutes (C), and 70 minutes (D) postsedation. Gallbladder wall thickening (white arrow) was first observed 30 minutes following sedation and was asymmetric (B), only seen along the dorsal (near field) aspect of the gallbladder wall. As time progressed, the ventral aspect of the gallbladder wall became asymmetrically thickened (C) until eventually the entire wall was diffusely thickened (D). Maximum gallbladder wall measurements depicted below were 1.7 mm, 2.8 mm, 3.4 mm, and 4.1 mm, respectively

Duration of sedation was significantly associated with thickening of the gallbladder wall (*P* < .001). Compared to baseline values (1.51 ± 0.25 mm), the gallbladder wall was significantly thicker (*P* ≤ .02) from 30 minutes (2.02 ± 0.22 mm) to the end of the observation period (2.73 ± 0.25 mm). The proportion of dogs developing gallbladder wall thickening in left lateral (5/10, 50%; 95% CI, 18.7%‐81.3%) and dorsal recumbency (7/10, 70%; 95% CI, 34.8%‐93.3%) did not differ significantly (*P* = .45). Of those that developed gallbladder wall thickening, 5 did so in both positions whereas 2 only did so in dorsal recumbency. All dogs that developed gallbladder wall thickening did so within 20 to 40 minutes of being sedated. The mean gallbladder wall thickness in dogs that developed gallbladder wall thickening (3.5 ± 0.57 mm) was approximately twice that of those that did not (1.53 ± 0.25 mm). Similar to phase 1, asymmetric thickening was seen first along the dorsal aspect of the gallbladder wall, regardless of recumbency position. Inconsistently, the thickening would spread to a more diffuse pattern as time progressed in some, but not all dogs (see Figure 1).

**FIGURE 2:**
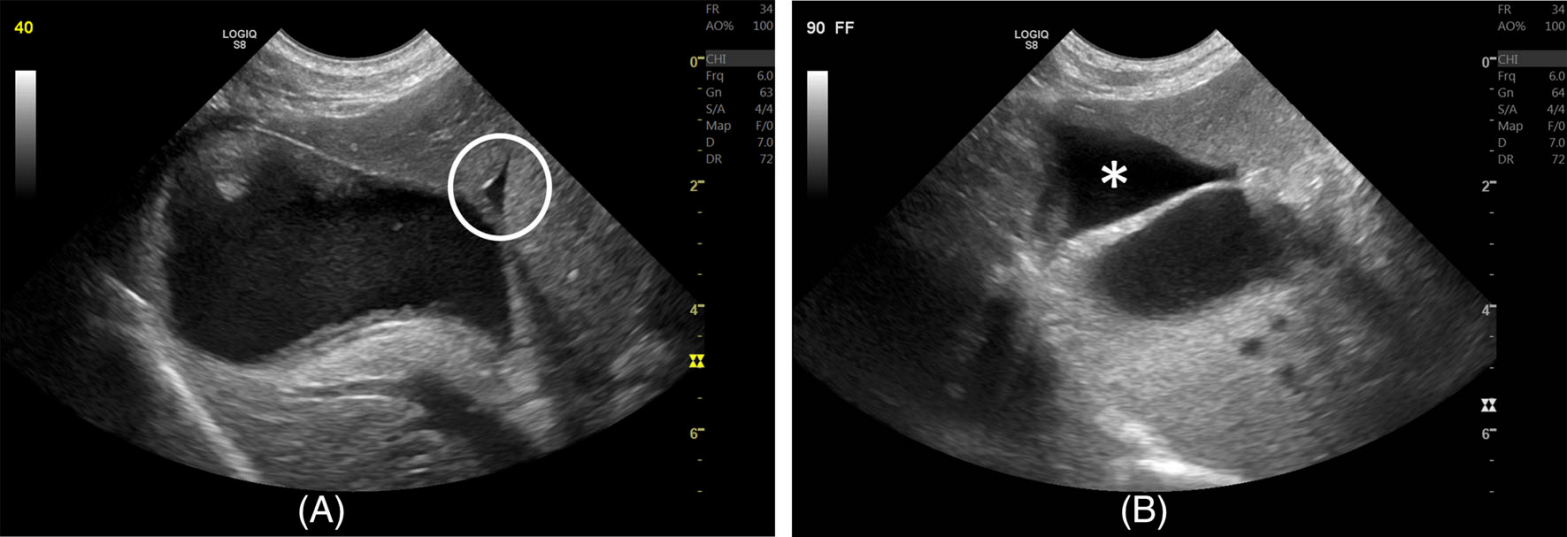
Two separate dogs from phase 2 of the study. (A) A small volume of peritoneal effusion (open circle) dissecting between the gallbladder wall and liver lobes 40 minutes after sedation with dexmedetomidine. (B) A moderate volume of peritoneal effusion (asterisk) dissecting between the gallbladder wall, liver lobes, and diaphragm 90 minutes after sedation

Unexpectedly, 5/10 (50%; 95% CI, 18.7%‐81.3%) dogs in lateral recumbency and 4/10 (40%; 95% CI, 12.2%‐73.7%) dogs in dorsal recumbency developed peritoneal effusion located between liver lobes, between liver lobes and the gallbladder, or between the caudal aspect of the liver and the stomach (see Figure 2). Some dogs developed effusion in both positions whereas others only in 1 position. Gallbladder wall thickness also was increased significantly in dogs with peritoneal effusion (*P* = .01). Four dogs had gallbladder sludge whereas 6 dogs did not. Secondary variables (see Table 2) not associated with gallbladder wall thickness included body weight, heart rate, systolic blood pressure, mean arterial blood pressure, presence of gallbladder sludge, and cross‐sectional area and blood flow velocities of the AO, caudal vena cava, and PV. All instances of gallbladder wall thickening persisted 10 minutes after reversal with atipamezole but resolved within 24 hours on follow‐up examination.

**TABLE 2:** Summary of variables measured in phase 2 to assess their association with the formation of gallbladder wall thickening in a population of 10 healthy research bloodhounds using separate linear mixed models with position within dog as a repeated effect and dog as a random effect

Variable	n	*P* value
Patient position	220	.45
Duration of sedation	220	<.001^a^
Peritoneal effusion	220	.009^a^
Gallbladder sludge	220	.44
Body weight	220	.06
Heart rate	209	.77
Systolic blood pressure	215	.64
Mean arterial blood pressure	215	.78
AO velocity	218	.97
CVC velocity	217	.75
PV velocity	218	.84
AO cross‐sectional area	217	.2
CVC cross‐sectional area	217	.59
PV cross‐sectional area	217	.88

Abbreviations: AO, aorta; CVC, caudal vena cava; PV, portal vein.

^a^ Significant association.

## 4 DISCUSSION

Our results support the primary hypothesis that sedation with dexmedetomidine alone, or in combination with other drugs, may cause transient gallbladder wall thickening in some dogs soon after sedation. This association has not been reported previously and is clinically relevant because benign gallbladder wall thickening could be confused with more serious pathologies if dexmedetomidine is given before ultrasonographic examination of the abdomen. Although our study was not designed to determine the exact time to resolution of the thickening, we did observe that some dogs in phase 2 took > 12 hours, but ≤24 hours for gallbladder wall thickness to return to normal. Hence, dexmedetomidine given hours before an ultrasound examination and unbeknownst to the sonographer could influence interpretation of the hepatobiliary region. In both study phases, the mean thickness of the gallbladder wall in dogs that developed thickening was approximately twice that of those that did not. The maximum thickness observed was 4.1 mm in phase 1 and 5.1 mm in phase 2. The prevalence in phase 1 was 24.1% with a median dexmedetomidine dose of 5.5 μg/kg and a median duration of sedation of 30 minutes. In contrast, the prevalence in phase 2 was 50% in left lateral recumbency and 70% in dorsal recumbency within 20 to 40 minutes of sedation with a dexmedetomidine dose of 10 μg/kg. Although subjective comparison of the prevalence between phase 1 and phase 2 suggests dexmedetomidine dose may play a role in the formation of gallbladder wall thickening, the data in phase 1 does not support a significant association. This result may represent type II error or a limitation of the study design. The effect of dose could be better investigated using a randomized, crossover design of escalating dosages of only dexmedetomidine in the same population of dogs to control for more variables.

The primary objective of phase 2 was to evaluate the effect of time and recumbency position on the formation of gallbladder wall thickening. As hypothesized, duration of sedation was positively associated with the thickness of the gallbladder wall. In all dogs in phase 2, the gallbladder wall was significantly thicker (*P* ≤ .02) from 30 minutes to the end of the observation period as compared to baseline results. This finding supports that dexmedetomidine causes thickening, even when the resultant gallbladder wall thickness does not exceed the upper end of the reference range of 2 mm. We hypothesized that dorsal recumbency would be significantly associated with gallbladder wall thickening, possibly because of the effects of abdominal venous compression on venous return or lymphatic drainage. This hypothesis was not supported by data from phase 2. Because of the small sample size (n = 10) and potential for a type II error, post hoc power analysis determined a follow‐up study with a sample size of 63 dogs would have sufficient power (β = 0.8; α = 0.05) to better evaluate the effect of position on gallbladder wall thickness.

One surprising finding from phase 2 was the observation of peritoneal effusion in 40% of dogs in lateral recumbency and 50% of dogs in dorsal recumbency. This also carries considerable clinical importance because any more than a trace amount of peritoneal effusion in adult dogs typically is pathologic and reported with numerous diseases that cause gallbladder wall thickening, including hepatitis, cholecystitis, pancreatitis, neoplasia, right‐sided heart failure, cardiac tamponade, and anaphylaxis. Unfortunately, our study was not designed to completely investigate this finding because only the hepatobiliary region of the abdomen was interrogated. The volume of fluid was not quantified but subjectively appeared small to moderate and often was seen dissecting between the liver lobes or between the stomach and caudal left liver. Additional studies could confirm an equal or higher frequency of peritoneal effusion by performing a full diagnostic ultrasound examination. The finding of peritoneal effusion highlights the species, regional anatomic, and pathophysiologic differences dexmedetomidine may exert because a recent study^9^ found that a dose of 50 μg/kg actually decreased the frequency of pleural effusion (and pulmonary edema) in a rat model of alpha‐naphthylthiourea‐induced acute lung injury.

The exact cause of gallbladder wall thickening remains unclear. Similar to most other pathologic causes of thickening, dexmedetomidine likely causes thickening through the formation of gallbladder wall edema. Because of ethical considerations, this hypothesis was not confirmed with histopathology. As with most tissues in the body, gallbladder wall edema likely is explained by aberrations in Starling's forces, including increased hydrostatic pressure, decreased lymphatic drainage, decreased oncotic pressure, or increased vascular permeability. It is well documented that dexmedetomidine causes bradycardia, increased systemic vascular resistance, and systemic hypertension, resulting in a decrease in cardiac output of up to 50%.^7^, ^10^, ^11^ Peripheral vasoconstriction also results in redistribution of blood to more vital organs.^7^ It is plausible that the combined cardiovascular effects of dexmedetomidine cause venous congestion via increased hydrostatic pressure and possibly decreased lymphatic drainage within the hepatobiliary system, resulting in the formation of gallbladder wall edema. It is unlikely that increased vascular permeability or a transient decrease in oncotic pressure would play a role.

In our study, numerous secondary variables were explored in an attempt to support an underlying pathophysiology for the formation of gallbladder wall thickening. All but 1 of the secondary variables were not associated with the gallbladder wall thickening. Contrary to the hypothesis, obesity and congestion index were not positively associated. In a recent study,^8^ obese dogs were shown to have lower mean portal velocity and PBFV as compared to dogs of ideal body condition. In obese humans, hepatocytes enlarge because of lipid droplets, which results in compression of hepatic vessels and hence increased vascular resistance and decreased compliance.^12^ Additionally, the weight and compressive effect of extraperitoneal fat stores could decrease venous and lymphatic drainage from the hepatobiliary region. However, in our population of dogs, the data do not support the potential for similar mechanisms of altered portal blood flow or decreased venous and lymphatic drainage from obesity causing gallbladder wall thickening. Paradoxically, increased PBFV was associated with not developing gallbladder wall thickening. One would expect that increased PBFV in the face of decreased cardiac output and decreased venous drainage via the caudal vena cava would increase hydrostatic pressure within the hepatobiliary system. The blood supply to the liver is 2‐fold with approximately 75% to 80% coming from the PV and the remainder supplied by the hepatic arteries.^13^ The increased PBFV may be offset by decreased blood supply via the hepatic arteries secondary to a dexmedetomidine‐mediated decrease in cardiac output. For example, 1 study^14^ found that dexmedetomidine caused a significant decrease in flow volume of the abdominal AO, the cranial mesenteric artery, and the celiac artery until reversal with atipamezole. However, it is also possible that the increased PBFV represents a statistically spurious result and is not truly clinically relevant because numerous secondary variables were analyzed.

Our study had some limitations. Given the prospective nature of phase 1, numerous variables could not be controlled. For example, the timepoint at which gallbladder wall thickening exceeded 2 mm was not observed. It is possible that some dogs developed thickening quickly, whereas others may have developed thickening exceeding 2 mm if observed for a longer period of time. A longer observation period may have changed the proportion of dogs with a thickness > 2 mm. However, it was not deemed ethical to keep dogs sedated longer than the imaging procedures required. The patient population was heterogeneous except for the fact that most were presented for orthopedic conditions. However, this heterogeneity also increases the generalizability of the results to a diffuse patient population that would likely be encountered in clinical practice. Phase 2 attempted to control numerous variables by use of a more homogeneous dog population, a constant dose, and the absence of other drugs. However, a study population of only 10 dogs introduces the potential for type II statistical error for many of the secondary variables because of a lack of power. Third, although both phases attempted to ensure patients did not have any underlying disease that could increase the risk for gallbladder wall thickening, it was neither financially nor ethically possible to conduct more extensive or invasive testing (eg, echocardiography, liver function testing, liver biopsy). Hence, we recognize the potential influence of some other unanticipated factor on the results of our study. However, the use of an appropriate history, physical examination, laboratory testing, and presonographic examination of the hepatobiliary system likely excluded most diseases or conditions that may have interfered with the results. Fourth, although every effort was made to collect complete data for all patients in phase 1, patient noncompliance during the presedation examination and beam attenuation secondary to gas within regional gastrointestinal structures precluded data collection for many secondary variables in some patients, most notably those involving the AO, caudal vena cava, and PV. Although the data collected in phase 2 was more complete, sample size was smaller. Finally, although the results of our study identify a clear association between the use of dexmedetomidine and gallbladder wall thickening in healthy dogs, it is unclear how other diseases (especially those of the hepatobiliary system) would influence the results. One would expect other diseases to increase the likelihood or severity of gallbladder wall thickening. However, perhaps certain inflammatory or fibrotic diseases would decrease the likelihood because of decreased tissue compliance arising from changes in the interstitium, vasculature, or lymphatics. For example, dogs with gallbladder sludge, the presence of which has been associated with decreased gallbladder emptying,^15^ did not have a positive association with gallbladder wall thickening after dexmedetomidine administration.

In conclusion, sedation with dexmedetomidine is associated with gallbladder wall thickening (> 2.0 mm) and peritoneal effusion in dogs undergoing abdominal ultrasonography which could be confused with pathologic etiologies. If possible, the hepatobiliary system should be evaluated before administration of dexmedetomidine. If not possible, the observation of mild to moderate thickening of the gallbladder wall (2‐5 mm) should be interpreted with caution and further contextualized with information from the patient's history, physical examination, and other imaging findings. Additionally, the gallbladder should be re‐evaluated no sooner than 24 hours after the administration of dexmedetomidine if thickening of the wall is seen.

## CONFLICT OF INTEREST DECLARATION

Authors declare no conflict of interest.

## OFF‐LABEL ANTIMICROBIAL DECLARATION

Authors declare no off‐label use of antimicrobials.

## INSTITUTIONAL ANIMAL CARE AND USE COMMITTEE (IACUC) OR OTHER APPROVAL DECLARATION

This project was approved by the Mississippi State University Institutional Animal Care and Use Committee: protocol numbers IACUC‐19‐024 and IACUC‐19‐026.

## HUMAN ETHICS APPROVAL DECLARATION

Authors declare human ethics approval was not needed for this study.
